# SARS-CoV-2 tetrameric RBD protein blocks viral infection and induces potent neutralizing antibody response

**DOI:** 10.3389/fimmu.2022.960094

**Published:** 2022-10-31

**Authors:** Zheng Liu, Chenglu Yang, Haokun Zhang, Guojie Cao, Senzhen Wang, Siwen Yin, Yanming Wang

**Affiliations:** Laboratory of Epigenetics and Translational Medicine, School of Life Sciences, Henan University, Kaifeng, Henan, China

**Keywords:** SARS-CoV-2, hACE2, 2xS-RBD-mFc, Vaccine, viral infection

## Abstract

The pandemic of coronavirus disease 2019 (COVID-19) caused by severe acute respiratory syndrome coronavirus 2 (SARS-CoV-2) has posed serious threats to global health and economy and calls for the development of safe treatments and effective vaccines. The receptor-binding domain in the spike protein (S^RBD^) of SARS-CoV-2 is responsible for its binding to angiotensin-converting enzyme 2 (ACE2) receptor. It contains multiple dominant neutralizing epitopes and serves as an important antigen for the development of COVID-19 vaccines. Here, we showed that dimeric S^RBD^-Fc and tetrameric 2xS^RBD^-Fc fusion proteins bind ACE2 with different affinity and block SARS-CoV-2 pseudoviral infection. Immunization of mice with S^RBD^-Fc fusion proteins elicited high titer of RBD-specific antibodies with robust neutralizing activity against pseudoviral infections. As such, our study indicates that the polymeric S^RBD^-Fc fusion protein can serve as a treatment agent as well as a vaccine for fighting COVID-19.

## Introduction

SARS-CoV-2, which causes the global pandemic coronavirus disease 2019 (COVID-19), belongs to a family of viruses known as coronaviruses that also include MERS−CoV and SARS-CoV-1 (1). Coronaviruses are commonly comprised of four structural proteins including spike protein (S), envelope protein (E), membrane protein (M) and nucleocapsid protein (N) (2). The SARS-CoV-2 S protein is a glycoprotein that mediates membrane fusion and viral entry. The S protein is homo-trimeric, with each ~180 kDa monomer consisting of two subunits S1 and S2 (3). In SARS-CoV-2, as with most coronaviruses, proteolytic cleavage of the S protein into S1and S2 subunits is required for activation (3). The S1 subunit mediates attachment of the S protein to the host receptor, while the S2 subunit is involved in cell fusion (4, 5). A receptor binding domain (RBD) in the C-terminus of the S1 subunit has been identified, and the RBD of SARS-CoV-2 shares 73% amino acid identity with the RBD of the SARS-CoV-1 but only 22% identity with that of MERS−CoV (6, 7). The low amino acid sequence homology is consistent with the finding that SARS and MERS−CoV bind different cellular receptors (8). The RBD of SARS-CoV-2 S protein binds angiotensin-converting enzyme 2 (ACE-2), a metallopeptidase, similar to that of SARS-CoV-1 but with much higher affinity and faster binding kinetics (9, 10). The SARS-CoV-2 Spike protein uses ACE2 to enter cells and the receptor-binding domains of SARS-CoV-2 Spike and SARS-CoV Spike bind with similar affinities to human ACE2 (11). Structural analysis of the S1 trimer shows that before binding to the ACE-2 receptor, only one of the three RBD domains is in the “up” conformation. This is an unstable and transient state that passes among trimeric subunits but is nevertheless an exposed state that can be targeted by neutralizing antibodies (12). For the potent antibodies, interacting with the RBDs are universally in the ‘up’ state, such full occupancy in each complex could render RBD completely inaccessible for ACE2 (13).

Polyclonal antibodies to the RBD of the SARS-CoV-2 protein have been shown to inhibit interaction with the ACE-2 receptor, confirming RBD as an attractive target for vaccinations and antiviral therapy (14). A single dose of AZD7442 had efficacy for the prevention of Covid-19 infection, without evident safety concerns (15). There is also promising work showing that the RBD may act as an antigen to interact with antibodies in a patient’s bloodstream, consistent with immunity developed after exposure to the SARS-CoV-2 (16). Several newly emerged SARS-CoV-2 variant genomes have been identified including the Omicron-B.1.1.529 variant. First identified in November 2021 in South Africa, this Omicron variant quickly became the dominant SARS-CoV-2 variant and is considered a variant of concern (VOC). The Omicron variant contains 15 mutations in RBD domain that potentially affect viral fitness and transmissibility. Most of the mutations are involved in ACE-2 binding leading to a higher Omicron and ACE-2 binding affinity, which potentially explains its much-increased transfection ability (17, 18). Several of these mutations facilitate immune escape and reduce neutralization activity of several monoclonal antibodies (17). mRNA, recombinant virus and inactivated virus vaccines have been developed and showed great efficacy (19, 20). Particularly, RBD-mRNA vaccine effectively protected mice from challenge with a virulent mouse-adapted SARS-CoV-2 variant (21). A self-amplifying RNA encoding the SARS-CoV-2 spike protein encapsulated within a lipid nanoparticle (LNP) as a vaccine showed remarkably high and dose-dependent SARS-CoV-2 specific antibody titers in mouse sera, as well as robust neutralization of both pseudo-virus and wild-type virus (22). However, the mRNA vaccine needs to be encapsulated: mRNA is unstable under physiological conditions, and the half-life of mRNA is short, and there may be problems or side effects beyond expectations (23). A clinical stage multivalent SARS-CoV-2 spike receptor-binding domain nanoparticle (RBD-NP) vaccine protects mice from SARS-CoV-2 challenge after a single immunization, indicating other potential strategies (24). Regardless of the type of vaccine, due to the in-time vaccination, current pandemic results show the sacrifices are lower than previously estimated (25).

However, even with the success of these vaccines, breakthrough infection still occurs at a decreased rate, and imposes a social and economic impact. For patients that are infected after vaccination, there is still a need to develop drugs to decrease symptoms and save lives. Here, we designed S^RBD^-Fc and 2xS^RBD^-Fc fusion proteins that can block pseudo virus infection and serve as immunogens to generate neutralizing antibodies. We engineered 293T cells with surface expression of ACE2, and expressed and purified S^RBD^-Fc and 2xS^RBD^-Fc fusion proteins. S^RBD^-Fc and 2xS^RBD^-Fc fusion proteins dimerize *via* Fc-mediated disulfide bond formation. We found that 2xS^RBD^-Fc dimer binds to ACE2 on the surface of 293T cells with an affinity 7-fold higher than that of the S^RBD^-Fc dimer. Both S^RBD^-Fc and 2xS^RBD^-Fc fusion proteins can block the infection of pseudo virus to ACE2 expressing 293T cells. We further showed that S^RBD^-Fc and 2xS^RBD^-Fc fusion proteins can generate antiserum with virus neutralizing activity in mice. Both fusion proteins can be produced in an industry-standard Chinese hamster ovary (CHO) cell system, suggesting this technology platform can scale up protein production, and promising for future drug design and clinical development to control the COVID-19 pandemic.

## Results

### Construction of HEK293T cells with constitutive expression of human ACE2

**Figure 1:**
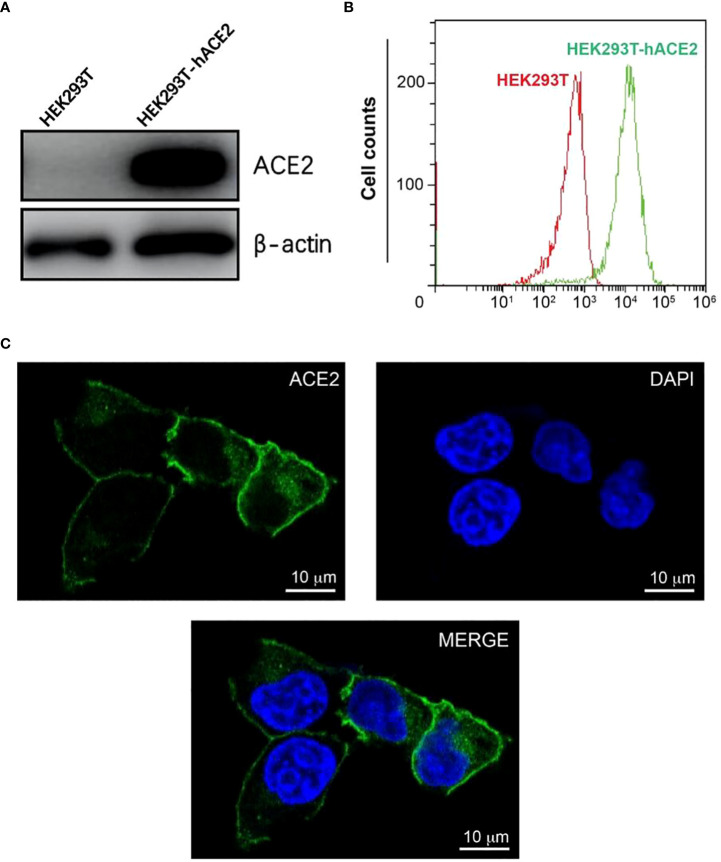
Construction and characterization of HEK293T-hACE2 cells. **(A)** Immunoblots show the expression of hACE2 in HEK293T-hACE2 cells but not HEK293T cells. β-actin was probed as a loading control. **(B)** Flow cytometry assays show the expression of hACE2 by the HEK293T-hACE2 cells compared to parental HEK293T cells after staining with an anti-hACE2 antibody. **(C)** Microscope images show the expression of human ACE2 in the HEK293T-hACE2 cells (DNA staining DAPI in blue, hACE2 antibody staining in green, scale bars 10 µm).

To create a host cell line that can be efficiently infected by pseudo viruses with the SARS-CoV-2 spikes, we transduced HEK293T cells with a lentiviral vector expressing human ACE2(hACE2) under an EF1a promoter. To create a founder cell line from the bulk transfection, we treated transfected cells with puromycin, diluted in 96 well plates, and re-expanded singular cells into a large population. We characterized an expanded clone that expressed high levels of hACE2 (**Figure 1A**). The hACE2 expression appeared stable over time and has not decreased noticeably after its establishment. This clone is hereafter referred to as HEK293T-hACE2.

We next tested the localization of hACE2 in the HEK293T-hACE2 cells. The expression of hACE2 on the surface of the membrane was observed by confocal microscopy using immunofluorescence staining with an anti-ACE2 antibody (**Figure 1C**). Additionally, flow cytometry analysis also detected hACE2 on the HEK293T-hACE2 cells without permeabilizing the cells (**Figure 1B**). These results indicate that hACE2 was appropriately localized to the membrane with its extracellular domain positioned in the right direction.

### Expression of recombinant S^RBD^-mFc and 2xS^RBD^-mFc fusion proteins

**Figure 2:**
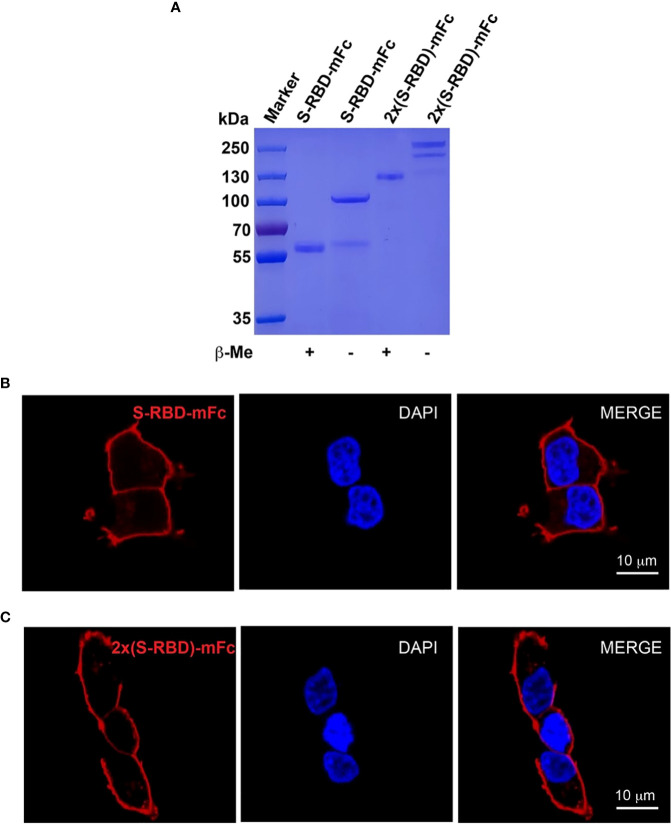
Expression and characterization of S^RBD^-mFc and 2xS^RBD^-mFc. **(A)** SDS-PAGE analysis of purified S^RBD^-mFc and 2xS^RBD^-mFc fusion proteins in reduced and non-reduced conditions, respectively. **(B, C)** Confocal microscope images show the S^RBD^-mFc and 2xS^RBD^-mFc binding to human ACE2 expressed on the HEK293T-hACE2 cell surface (DNA DAPI staining in blue, S^RBD^-mFc or 2xS^RBD^-mFc staining in red). Scale bars, 10 µm.

To obtain SARS-CoV-2 S^RBD^ (S protein receptor binding domain) and tandem 2xS^RBD^ proteins, we constructed two expression vectors encoding RBD and Fc fragment of mouse IgG (protein sequence in the supplementary). After expression in ExpiCHO cells and purification with affinity chromatography, we analyzed the S^RBD^-mFc and 2xS^RBD^-mFc proteins in both reduced and non-reduced conditions using SDS-PAGE. As shown in **Figure 2A**, we observed a single band with a molecular weight of ~57 kDa in the reduced condition and a band of ~115 kDa in the non-reduced condition, suggesting that the S^RBD^-mFc protein was well-expressed and purified with high purity, and formed a homodimer *via* the disulfide bond in the non-reduced condition. In addition, the 2xS^RBD^-mFc fusion protein had a molecular weight of ~100 kDa under reduced condition, and a molecular weight of ~200 kDa under non-reduced condition, indicating the disulfide bond mediated dimer formation through the Fc fragment.

Next, we investigated whether the S^RBD^-mFc and 2xS^RBD^-mFc proteins can bind to the surface of HEK293T-hACE2 cells. We found that the S^RBD^-mFc and 2xS^RBD^-mFc proteins could effectively bind to the hACE2 in a dose-dependent manner. As shown in **Figures 2B, C**, the receptor-ligand interaction of S^RBD^-mFc or 2xS^RBD^-mFc with hACE2 was observed with confocal microscope after immunofluorescence staining with DAPI and an anti-mouse IgG antibody. Moreover, the S^RBD^-mFc and 2xS^RBD^-mFc proteins were mainly observed on the surface of the cell membrane.

### S^RBD^-mFc and tandem 2xS^RBD^-mFc exhibited high binding affinity to hACE2

**Figure 3:**
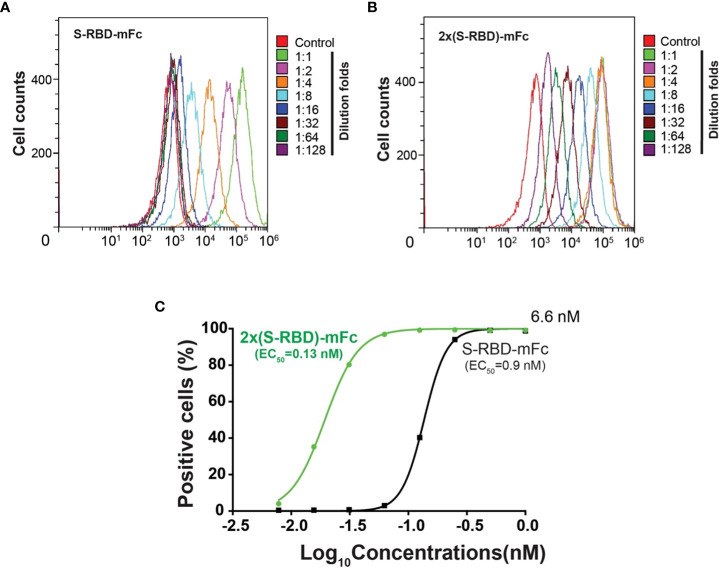
Affinity between hACE2 and the S^RBD^-mFc and the 2xS^RBD^-mFc. **(A, B)** Flow cytometry diagrams show the S^RBD^-mFc and 2xS^RBD^-mFc of binding to human ACE2 expressed on the HEK293T cell surface at a 2-fold series dilution. **(C)** Affinity binding curves for S^RBD^-mFc or 2xS^RBD^-mFc fusion proteins were established based on the flow cytometry results.

Using flow cytometry, we investigated the binding affinity between human ACE2 expressed on the HEK293T cell surface and the S^RBD^-mFc or the 2xS^RBD^-mFc fusion proteins (**Figures 3A, B**). We found that S^RBD^-mFc and 2xS^RBD^-mFc effectively bound to the hACE2 in a dose-dependent manner with a 50% effective binding concentration (EC_50_) at 0.9 nM and 0.13 nM, respectively (**Figure 3C**).

### S^RBD^-mFc and 2xS^RBD^-mFc block the infection of SARS-CoV-2 S protein pseudo virus

**Figure 4:**
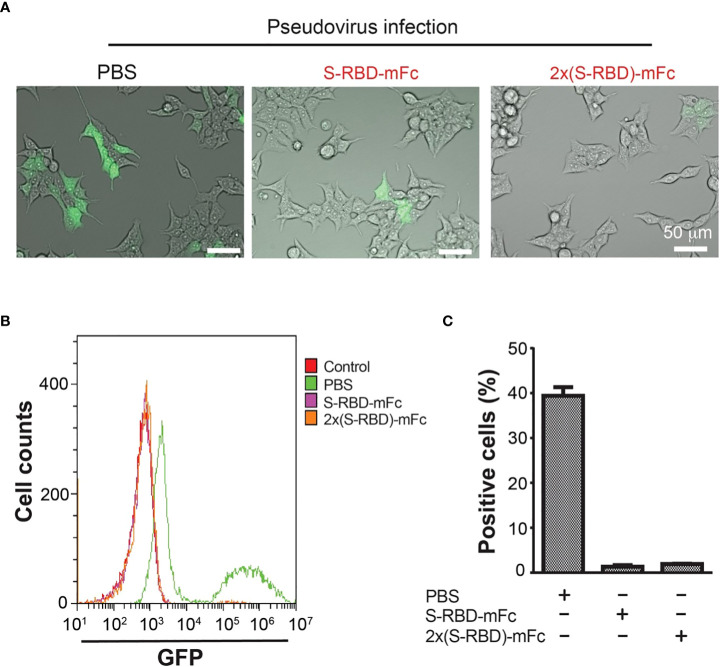
HEK293T-hACE2 cells infection by the SARS-CoV-2 S protein Pseudo virus was blocked by the S^RBD^-mFc and 2xS^RBD^-mFc fusion proteins. **(A)** Microscope images showing GFP expression in the HEK293T-hACE2 cells at 48h after incubation with SARS-CoV-2 Spike Pseudo virus premixed with PBS, S^RBD^-mFc and 2xS^RBD^-mFc. Scale bars, 50 µm. **(B)** Flow cytometry plots show cells of GFP expression. HEK293T-hACE2 cells without virus infection were analyzed as a negative control. Other cell groups were treated similar as described in **(A)**. **(C)** Mean values of cell percentages with high levels of GFP expression are shown (n = 3).

We next used the GFP-expressing SARS-CoV-2 S protein pseudo virus to analyze if S^RBD^-mFc and 2xS^RBD^-mFc proteins block the virus binding and entry into the HEK293T-hACE2 cells. The interaction of ACE2 with the S protein simulates pseudo viruses to enter and infect the target cells (26). We used the spike pseudo virus at 10-fold of the HEK293T-hACE2 cells. We first mixed pseudo virus with a high concentration of S^RBD^-mFc or 2xS^RBD^-mFc protein. We then added the mixture to a pre-seeded plate of HEK293T-hACE2 cells. As shown in **Figure 4A**, at 48 h post-infection, cells with green fluorescence signals indicating pseudo virus infection were much less after coincubation with S^RBD^-mFc or 2xS^RBD^-mFc fusion proteins than those after coincubation with PBS.

To quantify the efficiency of S^RBD^-mFc or 2xS^RBD^-mFc proteins in competition with the pseudo virus, HEK293T-hACE2 cells was incubated with a mixture of GFP-expressing SARS-CoV-2 S protein pseudo viruses premixed with S^RBD^-mFc or 2xS^RBD^-mFc proteins in a dilution series. After transfection, HEK293T-hACE2 cells were trypsinized and flow cytometry analyses were performed to analyze the green fluorescence signals and viral infection (**Figure 4B**). We found that cells infected with the pseudo virus alone showed stronger GFP signals with a second peak of much elevated signals accounting for 38% of all cells in this group (**Figures 4B, C**). In contrast, only low levels of green fluorescence signals were observed after infection of the pseudo virus premixed with the S^RBD^-mFc or 2x S^RBD^-mFc fusion proteins. Above results indicate that S^RBD^-mFc fusion proteins significantly blocked the infection of HEK293T-hACE2 cells by the pseudo virus.

### Neutralization assays with SARS-CoV-2 S protein pseudo virus

**Figure 5:**
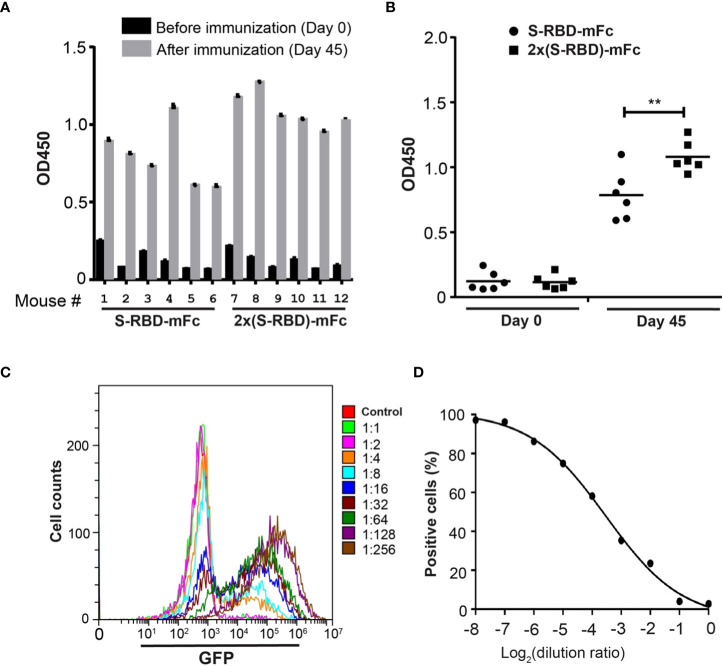
2xS^RBD^-mFc as a vaccine to generate neutralization antibodies. **(A)** ELISA assays of the anti-RBD antibody activity from antisera before and at day 35 after immunization. **(B)** The antibody activity in antisera immunized with S^RBD^-mFc is significantly less than that in the antisera immunized with 2xS^RBD^-mFc (**, P ≤ 0.01, n=6). **(C)** Flow cytometry diagrams show the GFP expression in HEK293T-hACE2 cells and HEK293T-hACE2 cells at 48h after infection with SARS-CoV-2 spike pseudo viruses premixed with mouse #8 antisera at a 2-fold dilution series. **(D)** Competition efficacy curve of antiserum #8 was established based on the flow cytometry results.

Antisera from mice after S^RBD^-mFc or 2x S^RBD^-mFc protein immunization were analyzed by ELISA using the His6-RBD fusion protein. Antisera collected after the last immunization showed much higher affinity than the pre-immune sera (**Figure 5A**). The antiserum from mouse 8 showed the strongest affinity for His6-RBD, and the titers of the antiserum based on dilution curves are shown in the **Supplementary Figure 1A**. These results indicate that S^RBD^-mFc and 2xS^RBD^-mFc act as effective immunogen to produce anti-RBD antibodies. Compared to mice immunized with S^RBD^-mFc, mice immunized with 2xS^RBD^-mFc exhibited an increased anti-RBD antibody activity (**Figure 5B**; **Supplementary Figure 1B**).

To evaluate the antiserum contains neutralizing antibodies against SARS-CoV-2, we performed viral infection experiments using the GFP-expressing SARS-CoV-2 S protein pseudo virus (27). The antiserum from mouse 8 neutralized pseudo virus infection at a concentration dependent manner (**Figures 5C, D**). To determine whether mutations in RBD are resistant to the neutralizing antibodies induced by 2xS^RBD^-mFc, we constructed pseudo virus with RBD of Omicron BA.4 S protein and analyzed its sensitivity to the neutralization activity of mouse antisera. At 48 h post-infection, HEK293T-hACE2 cells showed green fluorescence signals while the HEK293T cells did not, indicating that the Omicron BA.4 S protein pseudo virus infected cells by interacting with ACE2 (**Supplementary Figure 2A**). The antiserum from mouse 8 neutralized Omicron BA.4 pseudo virus infection at a concentration dependent manner (**Supplementary Figures 2B, C**). These results indicate that the antiserum could inhibit SARS-CoV-2 infection as well as other newly emerged variants with mutants in the spike protein.

## Discussion

Here, we designed and studied a tetrameric form of S RBD protein (2xS^RBD^-mFc) and a dimeric form of S RBD protein (S^RBD^-mFc). Since the S protein interacts with the ACE2 receptor as a trimer (12), we speculated that multimeric S-RBD proteins will have a higher binding affinity with the ACE2 receptor due to coordinated binding. Structural analysis of the S1 trimer shows that before binding to the ACE-2 receptor, only one of the three RBD domains is in the “up” conformation. Potent antibodies interact with the RBDs that are universally in the ‘up’ state, leading to full RBD occupancy in each complex and RBD completely inaccessible for ACE2. In addition, the neutralizing epitopes in SARS-CoV-2 RBD are conformational and nonlinear, which is consistent with those in SARS-CoV RBD (27). Fc fusion protein can form dimer through the disulfide bond of Fc hinge region, so that the dimeric and the tetrameric RBD-Fc fusion proteins can be easily formed by fusing one RBD domain or two tandem RBD domains to the N-terminal of the Fc fragment. The RBD domains of the RBD-Fc fusion protein also facilitate forming the exposed “up” RBD-domain conformation. A previous report has found that a disulfide-linked SARS-CoV-2 RBD dimer enhances the neutralizing antibody titer compared to the RBD monomer (28). Different from the disulfide-linked dimer approach, we fused the IgG1 Fc fragment to the C-terminus of SARS-CoV-2 RBD to produce dimeric and tetrameric S-RBD proteins, which indeed showed excellent binding capacity to hACE2. Compared with the dimeric form, the tetrameric form protein has elevated ability to block SARS-CoV-2 S protein pseudo virus infection, elicited higher neutralizing antibody titers in mice. We found that the tetrameric S RBD protein binds to ACE2 on the surface of 293T cells with an affinity 7-fold higher than that of the dimeric S RBD protein. The higher affinity between 2xS^RBD^-mFc and ACE2 likely correlates with the increased valency of binding sites between the 2xS^RBD^-mFc and the ACE2 trimer. Furthermore, both fusion proteins can block the infection of pseudo virus to 293T-hACE2 cells with high efficacy.

Fusion with Fc can increase the serum concentration of a target protein of interest, as well as generate high titers of neutralizing antibodies. A previous study has shown that the titer of neutralizing antibodies in the sera of mice immunized with Fc-fusion proteins was able to maintain at a high level for at least 3 months, generating antibody titers higher than that of neutralizing antibodies required for sterilizing immunity or full protection (27). The RBD-Fc-based SARS-CoV vaccine with V367F mutation showed enhanced binding affinity to ACE2 (29). The Fc fragment in RBD-based vaccine can serve as an immunopotentiator to enhance the immunogenicity of the vaccine since it promotes interaction of the vaccine with Fc receptor on the antigen-presenting cells (30). Importantly, the tetrameric S-RBD-Fc protein designed in our study has several advantages for increasing immunogenicity, such as increased molecular weight, better stimulation of immune cells and exposure of the immunodominant epitopes for neutralizing antibody binding. Since our experiment was conducted in mice, we used mouse Fc fragments to allow primary immunogenicity with the RBD region. In future human Fc-fusion protein drug design, human IgG1 Fc fragment shall be preferred. Using antiserum collected from mouse immunized with tetrameric form S RBD protein, we managed to neutralize the SARS-CoV-2 spike and Omicron BA.4 spike protein pseudo virus from infecting HEK293T-hACE2 cells, supporting that neutralizing antibodies have a broad-spectrum activity against virus variants.

Previous studies found that SARS-CoV-2 spike protein RBD domain was the majority target of neutralizing antibodies (31). The MERS-CoV S RBD-dimer significantly increased neutralizing antibody titers and protected mice against viral infection (32). Studies also found that a disulfide-linked dimeric form of MERS-CoV RBD also significantly enhanced the antibody response and neutralizing antibody titers compared to the conventional monomeric form (28). Vaccination of human ACE2 transgenic mice with RBD-Fc could effectively protect mice from the SARS-CoV-2 challenge (27). In our research, dimeric and tetrameric S-RBD-Fc proteins can also bind with a higher affinity to the ACE-2 protein which may serve as a therapeutic agent by blocking the hACE2 site of host cells to reduce virus infection. For vaccination purpose, it can also produce antibodies against the tertiary structure of RBD. Taken together, our results and those from others indicate that engineered RBD-Fc fusion proteins have a good potential to be further developed as effective treatments and broad-spectrum vaccines to prevent infection of the current SARS-CoV-2 and mutant variants that are emerging.

## Materials and methods

### Plasmid construction

The RBD and 2xRBD [RBD-(GGGGS-GGGGS-GGGGS)-RBD] vector constructs were synthesized by QINGKE Inc. in pcDNA3.4 with an N-terminal IgG kappa signal peptide and a C-terminal mouse IgG Fc fragment. The RBD sequence includes residues 319–541 of SARS-CoV-2 (GenBank accession number: NC_045512) spike protein. The hACE2 cDNAs were cloned into the pCDH-EF1a-mcs-(PGK-Puro) vector.

### Protein expression and purification

Genes encoding residues 319–532 of SARS-CoV-2 (GenBank accession number: QHD43416.1) spike protein fused with the genes of Fc in its N-terminal were inserted into the pcDNA3.4 plasmid. The recombinant expression plasmids were transfected into ExpiCHO cells, and then the cells were cultured for seven days. After that, cell culture supernatants were collected and purified using affinity chromatography. The purified recombinant proteins were analyzed using SDS-PAGE. Briefly, 4-20% Bis-Tris SDS-PAGE was used to separate the proteins, and then the proteins in the gel were stained using Coomassie Brilliant Blue to visualize the protein bands.

### Construction of hACE2 expression cell line

Lentivirus packaging was carried out by co-transfecting HEK293T cells (ATCC) with the hACE2 vector and two helper vectors (psPAX2 and pMD2.G) *via* Lipofectamine 2000 (Life Technologies,11668027). Two days later, lentivirus-containing supernatant was collected and used to infect the target HEK293T cells. Single clones were picked and expanded following antibiotic selection. All cell lines were cultured in DMEM supplemented with glutamine, 10% fetal bovine serum and penicillin/streptomycin. Cell lines were grown in a humidified incubator at 37°C with 5% CO_2_.

### Western blot

Whole cell lysates were made with RIPA buffer (Sigma, R0278-50ML). After centrifugation protein concentrations were measured by BCA protein assay (Thermofisher, 23227). For immunoblotting equal amounts of protein lysates were separated by SDS-polyacrylamide gel electrophoresis (PAGE), transferred onto PVDF membranes (Millipore, IPVH00010), blocked with 5% milk dissolved from powder and probed with the antibody (rabbit anti-human ACE2 antibody (Sino Biological, 10108-T24).

### Flow cytometry

Flow cytometry was used to detect the expression of hACE2 and the binding of S^RBD^ fusion proteins with HEK293T-hACE2 cells. Briefly, HEK293T-hACE2 cells were incubated with anti-human ACE2 antibody for 2 h, HEK293T cells were used as a control. Serial dilutions of S^RBD^-mFc and 2xS^RBD^-mFc proteins were prepared in EP tubes, diluted fusion protein was incubated with HEK293T-hACE2 cells for 2 h, HEK293T cells were used as a control. The following secondary antibodies (1:1000) were used to detect hACE2 and mFc fusion proteins respectively, including: Alexa Fluor 488 Goat anti-Rat IgG (Abcam, ab175473) and Alexa Fluor 647 Goat anti-mouse IgG (Abcam, ab150115).

### Immunofluorescence

HEK293T-hACE2 cells were cultured on the microscope cover glass in 24-well plates at the density of 1x10^5^ cells/well. After 24 h, cells were washed in PBS then fixed in 4% PBS-buffered formalin for 30 min, then washed with PBS and incubated with anti-human ACE2 antibody or S^RBD^-mFc and 2xS^RBD^-mFc proteins. Afterward, cells were stained with secondary antibodies including: Alexa Fluor 488 Goat anti-Rat IgG (Abcam, ab175473) or Alexa Fluor 568 Goat anti-mouse IgG (Abcam, ab175473) at 1:1000 dilution. After staining, cells were examined using a laser scanning confocal microscopy (Zeiss 880).

### Generation of anti- SARS-CoV-2 RBD antibodies

S^RBD^-mFc and 2xS^RBD^-mFc proteins were used to immunize mice and generate anti- SARS-CoV-2 RBD antibodies. In brief, Female BALB/c mice at age of 5–6 weeks were used and divided into two groups. Mouse 1 to 6 immunized with S^RBD^-mFc as antigen, and mouse 7-12 immunized with 2xS^RBD^-mFc as antigen. Pre-immune sera were prepared before starting the immunization. 10 µg antigen mixed with equal volume of complete Freunde’s adjuvant were used in the first injection. After that, immunization was conducted every two weeks with incomplete Freunde’s adjuvant and 3 days after the fourth immunization antisera were collected.

### Enzyme-linked immunosorbent assay

ELISA plates were coated with SARS-CoV-2 RBD with His6 tag at 0.02 μg/mL in carbonate buffer solution at 4°C overnight. After washing and blocking with 5% milk, antiserums diluted in 5% milk was added to each well. After incubation at 37°C for 4 h, plates were washed and incubated with goat anti-mouse IgG (H+L)/HRP (Jackson lab) for 2 h. After washing, TMB solution was added to the plates and incubated at 37°C for 20 min. Then the same volume 1.0 M H_2_SO_4_ was added to stop the reaction. Absorbance at 450 nm was measured by a microplate reader (synergy neo2).

### Production of omicron BA.4 spike pseudo virus

Pseudo virus was produced by co-transfection 293T cells with psPAX2, pNL4-3-GFP-T2A-Puro, and plasmids encoding Omicron BA.4 spike vector using Lipofectamine 2000 (Thermofisher, 11668019). The supernatants containing virus were harvested at 48 h post transfection, centrifuged at 800 g for 5 min to remove cell debris, and passed through 0.45 μm filter.

### Neutralization of pseudo virus

Neutralization assays were performed using antiserum 8. Serial dilutions of antiserum 8 were made with 10% heat-inactivated fetal bovine serum (Thermofisher, 16140071). SARS-CoV-2 spike pseudo virus expressing a GFP reporter gene (Genewiz: COVID-19-GFP) and Omicron BA.4 spike pseudo virus in 100 μl DMEM containing 3× 10^4^ infectious units was added to each tube and incubated for 2 h at 37°C, respectively. HEK293T-hACE2 cells were cultured in 96-well plates at the density of 2×10^3^ cells/well (33). Before transferring the contents of each tube to HEK 293T-hACE2 cells, polybrene (Sigma, TR1003E) was added to each tube to increase the efficiency of infection. HEK293T-hACE2 cells incubated with PBS were used as a control. Then the plate was incubated for 48 h at 37°C in a 5% CO2 incubator. After infection, cells were detached, centrifuged, and resuspended in PBS. Infection by the S protein Pseudo virus was quantified by flow cytometry.

## Data availability statement

The original contributions presented in the study are included in the article/**Supplementary Material**. Further inquiries can be directed to the corresponding author.

## Ethics statement

The animal study was reviewed and approved by Committee of Medical Ethics and Welfare for Experimental Animals, Henan University School of Medicine.

## Author contributions

ZL and YW wrote the manuscript with substantial input from all the authors. ZL , HZ and SY performed the protein expression. ZL, GC, and SW performed the cell culture experiments. ZL and CY performed the animal studies. All authors contributed to the article and approved the submitted version.

## Funding

Thanks for the startup fund from Henan University to Yanming Wang.

## Conflict of interest

The authors declare that the research was conducted in the absence of any commercial or financial relationships that could be construed as a potential conflict of interest.

## Publisher’s note

All claims expressed in this article are solely those of the authors and do not necessarily represent those of their affiliated organizations, or those of the publisher, the editors and the reviewers. Any product that may be evaluated in this article, or claim that may be made by its manufacturer, is not guaranteed or endorsed by the publisher.
