# Supplementary material for: SARS-CoV-2 tetrameric RBD protein blocks viral infection and induces potent neutralizing antibody response
Source: Front Immunol. 2022 Oct 31;13:960094. doi: 10.3389/fimmu.2022.960094 (PMC9659643; doi:10.3389/fimmu.2022.960094)
Supplement: Supplementary file 1 [file DataSheet_1.docx]

**Supplemental Information**

**SARS-CoV-2 Tetrameric RBD protein blocks viral infection and induces potent neutralizing antibody response**

Sequences of proteins used in this study

His6-S^RBD^

RVQPTESIVRFPNITNLCPFGEVFNATRFASVYAWNRKRISNCVADYSVLYNSASFSTFKCYGVSPTKLNDLCFTNVYADSFVIRGDEVRQIAPGQTGKIADYNYKLPDDFTGCVIAWNSNNLDSKVGGNYNYLYRLFRKSNLKPFERDISTEIYQAGSTPCNGVEGFNCYFPLQSYGFQPTNGVGYQPYRVVVLSFELLHAPATVCGPKKSTNLVKNKCVNFHHHHHH

S^RBD^-Fc

RVQPTESIVRFPNITNLCPFGEVFNATRFASVYAWNRKRISNCVADYSVLYNSASFSTFKCYGVSPTKLNDLCFTNVYADSFVIRGDEVRQIAPGQTGKIADYNYKLPDDFTGCVIAWNSNNLDSKVGGNYNYLYRLFRKSNLKPFERDISTEIYQAGSTPCNGVEGFNCYFPLQSYGFQPTNGVGYQPYRVVVLSFELLHAPATVCGPKKSTNLVKNKCVNFLECICTVPEVSSVFIFPPKPKDVLTITLTPKVTCVVVDISKDDPEVQFSWFVDDVEVHTAQTQPREEQFNSTFRSVSELPIMHQDWLNGKEFKCRVNSAAFPAPIEKTISKTKGRPKAPQVYTIPPPKEQMAKDKVSLTCMITDFFPEDITVEWQWNGQPAENYKNTQPIMDTDGSYFVYSKLNVQKSNWEAGNTFTCSVLHEGLHNHHTEKSLSHSPGK

2xS^RBD^-Fc

RVQPTESIVRFPNITNLCPFGEVFNATRFASVYAWNRKRISNCVADYSVLYNSASFSTFKCYGVSPTKLNDLCFTNVYADSFVIRGDEVRQIAPGQTGKIADYNYKLPDDFTGCVIAWNSNNLDSKVGGNYNYLYRLFRKSNLKPFERDISTEIYQAGSTPCNGVEGFNCYFPLQSYGFQPTNGVGYQPYRVVVLSFELLHAPATVCGPKKSTNLVKNKCVNFGGGGSGGGGSGGGGSRVQPTESIVRFPNITNLCPFGEVFNATRFASVYAWNRKRISNCVADYSVLYNSASFSTFKCYGVSPTKLNDLCFTNVYADSFVIRGDEVRQIAPGQTGKIADYNYKLPDDFTGCVIAWNSNNLDSKVGGNYNYLYRLFRKSNLKPFERDISTEIYQAGSTPCNGVEGFNCYFPLQSYGFQPTNGVGYQPYRVVVLSFELLHAPATVCGPKKSTNLVKNKCVNFLECICTVPEVSSVFIFPPKPKDVLTITLTPKVTCVVVDISKDDPEVQFSWFVDDVEVHTAQTQPREEQFNSTFRSVSELPIMHQDWLNGKEFKCRVNSAAFPAPIEKTISKTKGRPKAPQVYTIPPPKEQMAKDKVSLTCMITDFFPEDITVEWQWNGQPAENYKNTQPIMDTDGSYFVYSKLNVQKSNWEAGNTFTCSVLHEGLHNHHTEKSLSHSPGK

**Red color:** SARS-CoV-2 spike protein, position 319-541

**Supplementary Figure. 1**


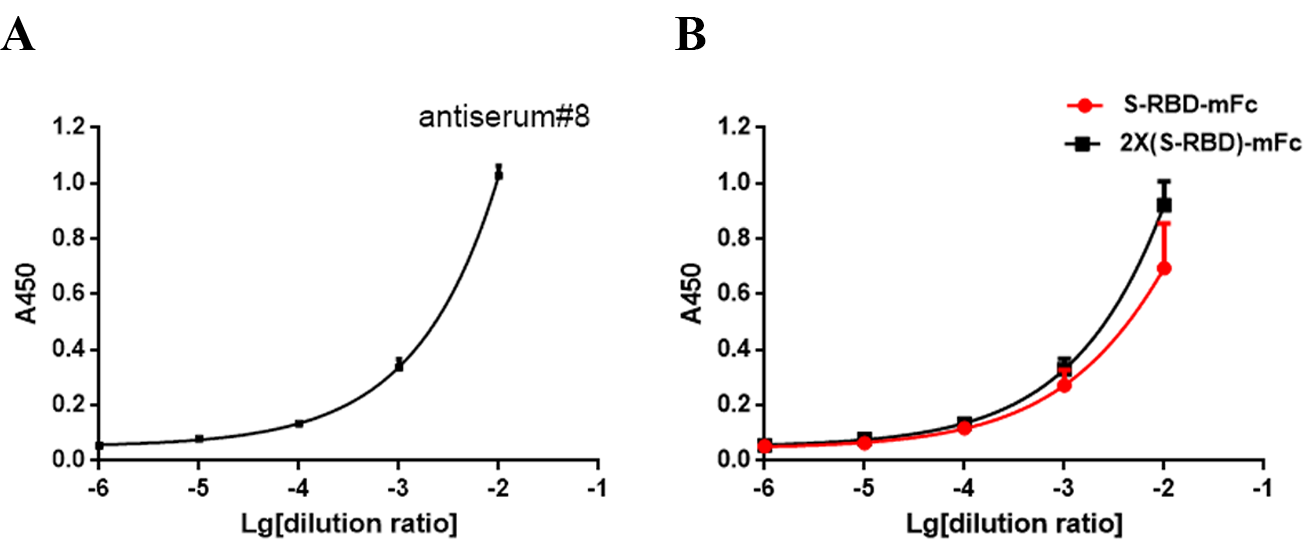


**Fig. 1 2xS^RBD^-mFc as a vaccine to generate neutralization antibodies.**

**(A)** ELISA assays of the anti-RBD antibody activity from antiserum#8. **(B)** The antibody activity in antisera immunized with S^RBD^-mFc is less than that in the antisera immunized with 2xS^RBD^-mFc (n=6). The A450 of antiserum in mice immunized 2xS^RBD^-mFc was about 1.2-fold higher than that of antiserum in mice immunized with S^RBD^-mFc (P<0.05, n=6).

**Supplementary Figure. 2**


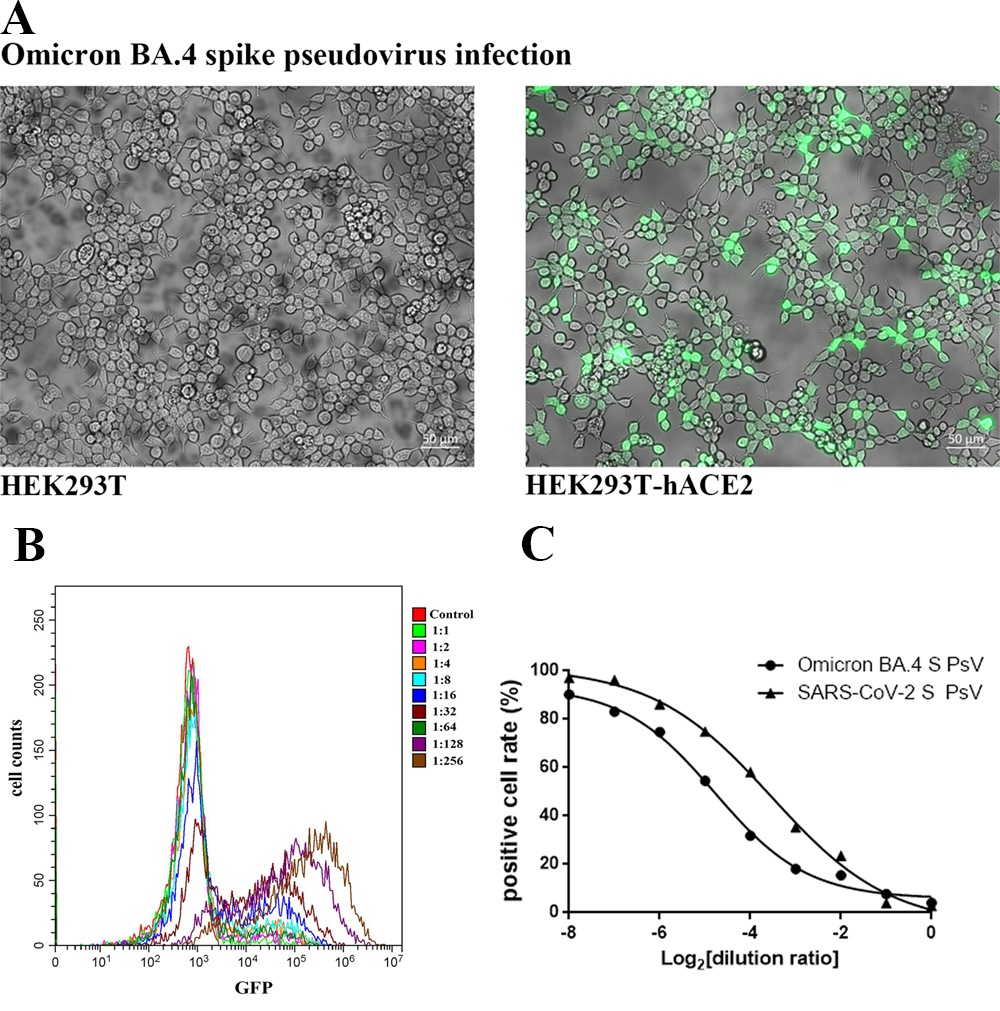


**Fig. 2 Neutralization activities of serum#8 against Omicron BA.4 spike pseudovirus**

**(A)** Microscope images showing GFP expression in the HEK293T and the HEK293T-hACE2 cells at 48h after incubation with Omicron BA.4 Spike Pseudovirus. Scale bars, 50 µm.**(B)** Flow cytometry diagrams show the GFP expression in HEK293T-hACE2 cells and HEK293T-hACE2 cells at 48h after infection with Omicron BA.4 spike pseudoviruses premixed with mouse #8 antisera at a 2-fold dilution series. **(C)** Competition efficacy curve of antiserum #8 was established based on the flow cytometry results.
